# Sensing Psychological Well-being Using Social Media Language: Prediction Model Development Study

**DOI:** 10.2196/41823

**Published:** 2023-01-31

**Authors:** Nuo Han, Sijia Li, Feng Huang, Yeye Wen, Xiaoyang Wang, Xiaoqian Liu, Linyan Li, Tingshao Zhu

**Affiliations:** 1 Chinese Academy Sciences Key Laboratory of Behavioral Science Institute of Psychology Chinese Academy of Sciences Beijing China; 2 Department of Psychology University of Chinese Academy of Sciences Beijing China; 3 School of Data Science City University of Hong Kong Hong Kong SAR Hong Kong; 4 Department of Social Work and Social Administration The Unversity of Hong Kong Hong Kong SAR Hong Kong; 5 School of Electronic, Electrical and Communication Engineering University of Chinese Academy of Sciences Beijing China; 6 Department of Infectious Diseases and Public Health Jockey Club College of Veterinary Medicine and Life Sciences City University of Hong Kong Hong Kong SAR Hong Kong

**Keywords:** mental health, psychological well-being, social media, machine learning, domain knowledge, mental well being, mental wellbeing, linguistic, predict, model, ground truth, lexicon

## Abstract

**Background:**

Positive mental health is arguably increasingly important and can be revealed, to some extent, in terms of psychological well-being (PWB). However, PWB is difficult to assess in real time on a large scale. The popularity and proliferation of social media make it possible to sense and monitor online users’ PWB in a nonintrusive way, and the objective of this study is to test the effectiveness of using social media language expression as a predictor of PWB.

**Objective:**

This study aims to investigate the predictive power of social media corresponding to ground truth well-being data in a psychological way.

**Methods:**

We recruited 1427 participants. Their well-being was evaluated using 6 dimensions of PWB. Their posts on social media were collected, and 6 psychological lexicons were used to extract linguistic features. A multiobjective prediction model was then built with the extracted linguistic features as input and PWB as the output. Further, the validity of the prediction model was confirmed by evaluating the model's discriminant validity, convergent validity, and criterion validity. The reliability of the model was also confirmed by evaluating the split-half reliability.

**Results:**

The correlation coefficients between the predicted PWB scores of social media users and the actual scores obtained using the linguistic prediction model of this study were between 0.49 and 0.54 (*P*<.001), which means that the model had good criterion validity. In terms of the model’s structural validity, it exhibited excellent convergent validity but less than satisfactory discriminant validity. The results also suggested that our model had good split-half reliability levels for every dimension (ranging from 0.65 to 0.85; *P*<.001).

**Conclusions:**

By confirming the availability and stability of the linguistic prediction model, this study verified the predictability of social media corresponding to ground truth well-being data from the perspective of PWB. Our study has positive implications for the use of social media to predict mental health in nonprofessional settings such as self-testing or a large-scale user study.

## Introduction

Many public health campaigns and “wellness” initiatives aim to promote positive mental health. Positive mental health is arguably increasingly important for public policy, the academic world, and popular imagination [1]. In the view of previous research, positive mental health has 3 core aspects: hedonic well-being and the psychological and societal aspects of eudaimonic well-being [2,3]. This study targeted one aspect of positive mental health: psychological well-being (PWB). The definition of PWB includes the criteria of liking most parts of one’s own personality, being good at managing the responsibilities of daily life, having positive relationships with others, and being satisfied with one’s own life [4-6]. In summary, PWB can reflect people's well-being to a certain extent and can also be used to evaluate people's positive mental health [7]. Moreover, studies have found that media use is linked to lower PWB [8], so it is essential in modern society to sense people’s PWB in time so as to provide a targeted intervention.

However, gauging people’s PWB is difficult in real time on a large scale, not only because of a lack of medical resources and staff but also due to the stigma associated with experiencing problems with mental health. In addition to explicit scales, researchers and practitioners encourage the use of passive techniques to address the imbalance between the demand and supply of mental health resources. For example, many studies have used individual physiological [9,10] and behavioral [11-13] indicators as predictors to help identify individuals with mental health problems.

Social media shows the potential to function as a viable “passive sensor” of mental health. Given the prevalence of social media, users generate a tremendous volume of content every day; social media can serve as a fountainhead of nonintrusive, real-time, and massive data that can be used to infer well-being [14]. The personal and social discourses that users follow on a daily basis make up the expression on social media, which can effectively reflect users’ health status and PWB in various contexts [15-17]. Thus, researchers are able to study a variety of mental illnesses, including depression, anxiety, stress, and loneliness [13,15,16,18-20], based on linguistic cues on social media platforms.

Although the capability of social media data has proved enormous, its predictive power corresponding to ground truth well-being data has yet to be endorsed [15]. By verifying whether social media expressions can reflect an individual's PWB, this study aimed to investigate this previously unconfirmed topic. Unlike previous studies that only used statistical methods to verify the accuracy of the prediction model, this study tested the reliability and validity of the model following the methods of psychological verification measurement tools. Thus, this study could further explain some indicators in psychology and validate the effectiveness of using a machine learning method to measure mental health. The data used in this study were collected from Sina Weibo—a leading Chinese social media platform featuring more than 926 million registered users [21]. This study targeted online active social media users and hypothesized that psychological traits, which are related to PWB, could be beneficial to establish a PWB prediction model. Therefore, we used psycholinguistic and psychological lexicons with corresponding traits related to PWB to extract linguistic features and ground truth PWB data to develop a machine learning model. We also validated the prediction model by referencing reliability and validity tests for psychological scales. Our study has positive implications for the use of social media to predict well-being in nonprofessional online settings such as a large-scale user study or user self-monitoring.

## Methods

### Participants

**Figure 1:**
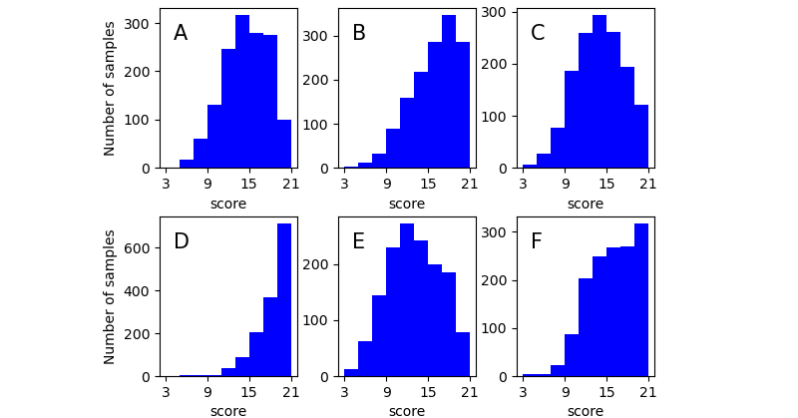
Prenormalized scores: (A) environment mastery, (B) positive relations, (C) autonomy item, (D) personal growth, (E) self-acceptance, (F) purpose in life.

**Figure 2:**
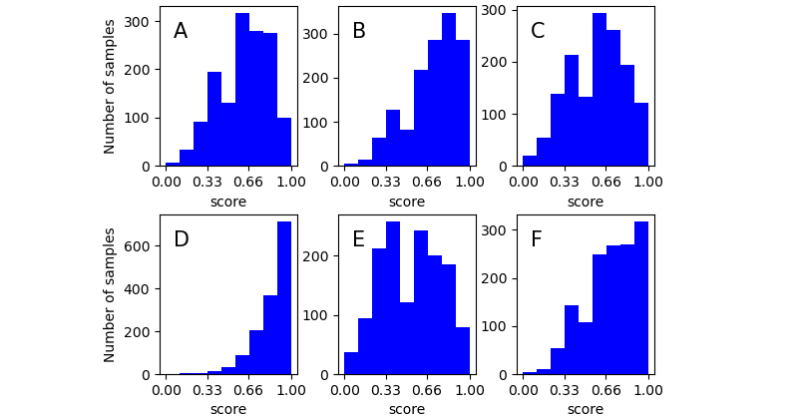
Normalized scores: (A) environment mastery, (B) positive relations, (C) autonomy item, (D) personal growth, (E) self-acceptance, (F) purpose in life.

In this study, a self-developed, online, experimental platform was used to recruit participants. We first randomly sent invitations to approximately 20,000 Weibo users whose profiles indicated they were adults. Then, users who were willing to participate in our experiment were guided to the online experimental platform. Between September 2012 and February 2013, a total of 2428 Weibo users participated in the experiment. To ensure that the participants were active users of Weibo and that the psychological questionnaire was valid, we excluded (1) participants who had posted fewer than 500 Weibo texts since the initial registration of personal accounts and (2) participants who completed the questionnaire over too long or too short a period of time. Specifically, considering that PWB was part of the entire collected questionnaire (18 of 72 questions), we excluded participants who completed the entire questionnaire in less than 300 seconds or more than 1500 seconds (during the pre-experiment, the total time to complete the questionnaire was 6 minutes to 15 minutes). After normalizing the PWB score distribution (the changes in the label set’s distribution are shown in Figures 1 and 2), 1427 samples were ultimately included in this study, including 540 men and 887 women. In the end, a total of 471,173 posts were included in the analysis of this study.

### Ethics Approval

The ethical conduct of the research was approved by the Institutional Review Board at the Institute of Psychology, Chinese Academy of Sciences, under code H15009.

### Instruments

#### Online Experimental Platform

**Figure 3:**
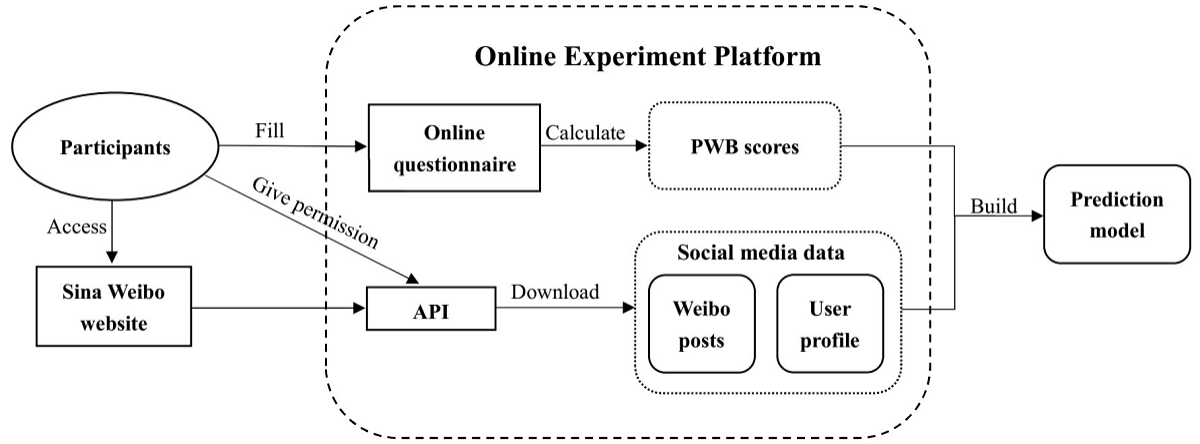
Information collection process via the online experiment platform. PWB: psychological well-being.

The online experimental platform provided the informed consent form; retrieved social media data from the participants, as discussed in the previous section; and included information on user profiles, their posts, and their online questionnaire results. The information collection process via the online experiment platform is shown in Figure 3. Referring to the ethical principles proposed by Kosinski et al [22], all users' privacy was strictly guaranteed in this process.

#### Psychological Well-being Scale

The PWB scale by Ryff and Keyes [23] is a theoretically grounded instrument that specifically focuses on measuring multiple facets of PWB. The reliability and validity of this PWB scale have been validated in long-term practice by numerous studies. The scale includes the following 6 facets: autonomy, environmental mastery, personal growth, positive relations with others, purpose in life, and self-acceptance [23]. Considering that the quality of the online questionnaire depends on the participants' conscious compliance with the rules for completing the questionnaire, we used the 18-item version of the PWB scale [7], which requires less time from participants. A brief description of the 6 dimensions is provided in Table 1. Participants evaluated themselves with respect to each question on a 7-point Likert scale, with answers ranging from strongly disagree (1 point) to strongly agree (7 points). Since each dimension contains 3 items, the participants' PWB scores for each dimension ranged from 3 to 21.

**Table 1:** Brief descriptions of the 6 dimensions of the PWB scale by Ryff and Keyes [23]. PWB: psychological well-being.

Dimension	Description
Environmental mastery	The ability to manage complex environments to suit personal needs and values
Positive relations with 0thers	The establishment of quality ties to others
Autonomy	A sense of autonomy in thought and action
Personal growth	Continued growth and development as a person
Self-acceptance	A positive attitude towards self
Purpose in life	The pursuit of meaningful goals and a sense of purpose in life

#### Linguistic Lexicons

This study focused on the ability to predict PWB based on participants' language habits, and in addition to the Linguistic Inquiry and Word Count (LIWC) dictionary commonly used in psycholinguistics, we used a series of psychological lexicons to extract features that were expected to be effective in predicting PWB. Specifically, we used the Simplified Chinese version of the LIWC (SC-LIWC) [24], Weibo Basic Mood Lexicon (Weibo-5BML) [25], Chinese Suicide Dictionary (CSD) [26], Moral Motivation Dictionary (MMD) [27], Moral Foundations Dictionary (MFD) [28], and Culture Value Dictionary (CVD) [29]. An introduction to these dictionaries and our reasons for choosing them are provided in the following paragraphs.

The LIWC is widely used in natural language processing for mapping the psychological and linguistic dimensions of written expression. In this research, we used the SC-LIWC, which reports 87 dimensions of language use in simplified Chinese [24]. This lexicon includes dimensions related to linguistic processes (eg, “swear words” and “common verbs”), psychological processes (eg, “anxiety words” and “health words”), personal concerns (eg, “achievement words” and “religion words”), and spoken categories (eg, “assent words” and “nonfluencies”) [30]. The SC-LIWC has been validated with respect to the detection of psychological expressions in short texts on social media [31]. Several studies have used the LIWC or SC-LIWC to construct computational prediction models of psychological traits. For example, scientists have been able to predict users’ personalities [19,32], mental health status [15,33], subjective well-being [17,34], and other factors based on textual data drawn from social media.

Weibo-5BML is a lexicon that contains 818 Chinese words or phrases that are annotated with 5 emotions (happiness, sadness, anger, fear, and disgust) [25]. The Weibo-5BML lexicon has been used to identify mood changes of Weibo users, and the reliability of this lexicon has been verified [35]. The intrinsic principle for including a sentiment lexicon (Weibo-5BML) is that people with different levels of PWB tend to express their well-being differently and hence use different words (phrases) to express different emotions. The association between emotions and PWB has also been observed in earlier research [36].

The CSD is intended to identify suicide risk on social media. This lexicon can be used not only to detect suicidal expressions in social media posts but also to evaluate levels of individual suicide risk. The CSD is composed of 2168 words that fit into 13 different categories (eg, “suicide ideation words,” “hopeless words,” and “personality words”) [26]. Li et al [37] used the CSD to measure users’ suicidal ideation risk on Weibo, which means that the CSD is reliable. We selected this lexicon because studies have found that a higher level of PWB is related to a negative attitude toward suicide [38].

The dictionary we used in our study, MMD, was developed by Zhang and Yu [27] in simplified Chinese. It includes 690 agency words and 260 communion words. The MMD has been used to measure the moral motivations of groups on social media by many psychological studies, such as the study by Zhao et al [39]. The reason we chose this dictionary is that a previous study has shown that integrated moral motivation is a crucial determinant of highly moral behavior and personal well-being [40].

The MFD can reflect the extent to which people follow basic moral norms in terms of their language habits [28]. This study used the simplified Chinese version of the MFD, which contains 590 Chinese words or phrases across 6 dimensions (harm, fairness, ingroup, authority, purity, and general morality). In addition to general morality, the other 5 dimensions all contain positive and negative word lists. Therefore, the MFD contains a total of 11 categories [41]. This dictionary has been used to measure group moral foundations in social media studies [37,42]. Many studies have suggested that moral behavior and moral thoughts are all effective means of boosting well-being [40,43].

The CVD consists of 53 individualistic Chinese words, 64 collectivistic Chinese words, and their synonyms [29]. This dictionary has been used to measure the spectrum of individualism-collectivism by previous psychological studies, such as those by Han et al [44] and Huang et al [45]. PWB, as a dimension of mental health [46], is clearly influenced by culture [5]. Therefore, in this study, we chose the spectrum of individualism-collectivism, which is an important expressive dimension of cultural value, to extract features for the purpose of predicting PWB.

### Procedures

#### Data Collection

First, we randomly sent invitations to approximately 20,000 Weibo users. Subsequently, users who were willing to participate in our experiment were instructed to use our online experiment platform to provide informed consent and complete a psychological questionnaire so that we could access their PWB scores. Finally, these users’ social media data (Weibo posts and user profiles) were downloaded via the Sina Weibo application programming interface (as shown in Figure 3).

#### Data Preprocessing

Subscale scores on the PWB scale by Ryff and Keyes were calculated after data collection. Subsequently, the total number of Weibo posts by all participants was counted, and only participants with more than 500 Weibo posts were retained for reference by this study. Then, considering that PWB is a relatively stable psychological variable, it is easily affected by life events and fluctuates. In order to exclude the random influence of life events, we split the posts into half by time instead of randomly drawing posts. For all remaining participant, we sorted all posts by each person in the order in which they had been posted. Next, we separately merged the sorted odd-numbered posts by each participant into 1 document, sorted even-numbered posts by each participant into 1 document, and all posts by each participant into 1 document. Finally, we had 3 documents, each document containing 1427 samples. We named the documents containing the odd-numbered and even-numbered posts “split-half” data, while the document containing all posts was termed “whole” data.

#### Feature Extraction

**Figure 4:**
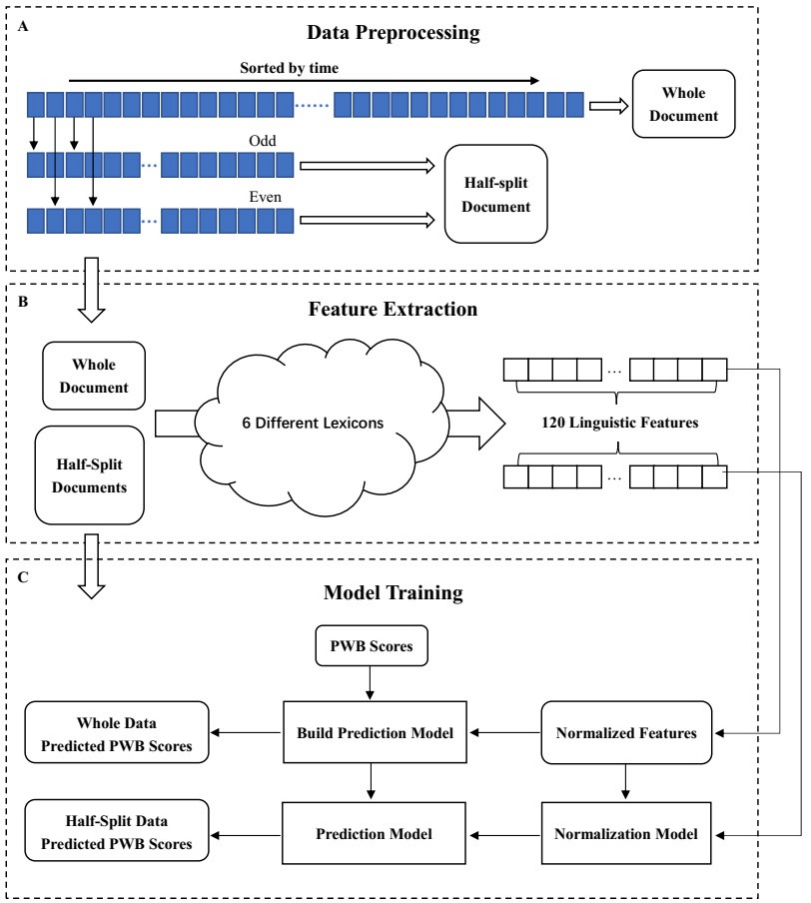
Data preprocessing, feature extraction, and model training. PWB: psychological well-being.

As shown in Figure 4, for “whole” data and “split-half” data, we extracted 120 dictionary-based linguistic features from each document (for the dictionary selection rules, please see the “Linguistic Lexicons” section). In detail, these linguistic features included 87 LIWC features, 5 Weibo-5BML features, 13 CSD features, 2 MMD features, 11 MFD features, and 2 CVD features*.*

Referring to the calculation method for language features, we first divided each document into several word pieces and then calculated the frequency of word pieces from each lexicon category as a language feature. Equation 1 shows the specific word frequency calculation method, in which context represents the i-th language feature, represents the j-th document, is the frequency with which the term in the i-th language category appears in the document, is the word count of the j-th document, and is the value of the i-th linguistic feature in the j-th document. Therefore, the larger the value is, the more frequently the i-th language feature occurs in the j-th document. Following feature extraction, a feature file was created with each row representing a sample and each column representing a linguistic feature. Specifically, for either “whole” data or “split-half” data, since we collected 1427 samples, the 3 feature files each contained 120 columns and 1427 rows.



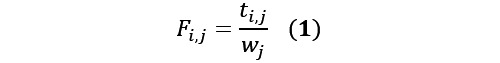



#### Model Training

During the first step of model training, all features were normalized to ensure that the feature contributions to the model were not affected by their range and distribution. Given that the tree model has a built-in feature selection function, no feature selection nor feature reduction was performed to retain as much information as possible. Although some categories may have had some of the same words, this would not have impacted the prediction because it did not add new information [47]. To make sure that the prediction model was not overfitted, 5-fold cross-validation was applied in the model validation.

After feature normalization, we got a sample size of 1427 with 120 features for each sample. We then used multiobjective extra trees (MOET) to develop a regression prediction model. As an extension of the random forest regression model [48], the extra trees algorithm was proposed as a computationally efficient and highly randomized extension of the random forest algorithm. The extra trees algorithm is an important algorithm within the class of decision tree–based ensemble learning methods and has been shown to have state-of-the-art performance on many regression tasks featuring high-dimensional inputs and outputs [49]. We used MOET because multiobjective learning can use multiple object modeling strategies to improve performance beyond the level of single object learning in the same scene [12]. During training, a MOET regression model with 1000 trees was trained using pairs of input linguistic features and annotated output PWB subscale scores.

**Figure 5:**
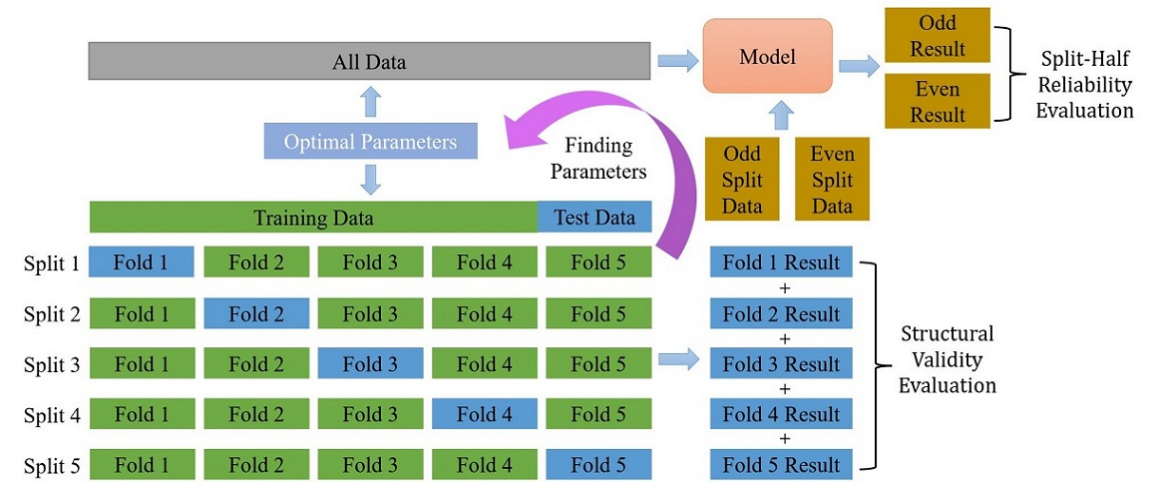
Data preservation rules and model evaluation.

The model parameters were tuned using 5-fold cross-validation. This method can better indicate the stability of the model on small samples, to make better use of samples. The prediction scores to assess the validity of the prediction model were also kept in this step. Roughly speaking, we randomly divided the sample into 5 equal parts and then, in each fold, selected 1 part as the test set and the remaining 4 parts as the training set. Finally, for all samples, when they were included in the test set, their predicted scores and corresponding ground truth scores were sequentially saved. Accordingly, all samples in the “whole” data were predicted once as test sets (see Figure 5). What we ended up using for the statistical analysis was the pooling of the 5 test set predictions produced by the 5-fold cross-validation.

Next, we developed a multiobjective prediction model of 120 linguistic dimensions using the “whole” data and then fitted the “split-half” data to the prediction model. Based on 1 set of “whole” data and 2 sets of “split-half” data, we obtained a total of 3 sets of predicted scores. Figure 4 depicts the overall process of data preprocessing, feature extraction, and model training.

### Statistical Analysis

In this study, we used multiobjective regression to consider all PWB dimensions as a whole to develop a novel model. However, a basic premise of this approach is that these dimensions are correlated (even weakly) [12]. Therefore, we first analyzed the Pearson correlation coefficients between PWB dimensions. Next, considering that we introduced domain knowledge to build the prediction model, we analyzed the feature importance of each psychological lexicon after using the attribute of the extra trees algorithm to output the feature importance of the language features that were used for modeling. In detail, we calculated the sum of the feature importance and the average feature importance (the ratio of the sum of the feature importance of each lexicon compared with the number of features included in each lexicon) of each lexicon. Last, the method by Wang et al [11] was used as a reference to verify the reliability and validity of the model. The split-half reliability for the prediction model was assessed between the “split-half” scores using Pearson correlation coefficients. Multitrait-multimethod matrix analysis and criterion validity analysis were conducted on the “whole” data to test the model’s validity [11]. All the Pearson correlation coefficients were calculated using SPSS 22.0 (IBM Corp, Armonk, NY) [50].

## Results

### Demographic Information

First, we collected demographic information from the participants. Among all the participants, 62.2% (887/1427) were women. Participants' ages ranged from 18 years to 80 years, with a median age of 23 (SD 5.8) years. Most of the participants were located in cities (1318/1427, 92.4%). Among the study population, 52.1% (743/1427) of the participants had a bachelor’s degree, and 41.4% (591/1427) of them had a high school degree or less. The vast majority (1305/1427, 91.4%) of the number of posts by participants were concentrated between 1000 and 5000 posts. Table 2 displays the demographic information of these individuals.

**Table 2:** Demographic information of the participants (n=1427).

Demographic information		Participants, n (%)
**Gender**		
	Male	540 (37.8)
	Female	887 (62.2)
**Age (years)**		
	18-30	1178 (82.6)
	31-40	108 (7.6)
	≥41	32 (2.2)
	Missing data	109 (7.6)
**Location**		
	Urban	1318 (92.4)
	Rural	109 (7.6)
**Educational background**		
	High school	591 (41.4)
	Bachelor’s degree	743 (52.1)
	Master’s degree	79 (5.5)
	Doctorate	10 (0.7)
	Missing data	4 (0.3)
**Post count**		
	500-1000	31 (2.2)
	1000-5000	1305 (91.4)
	≥5000	91 (6.4)

### Linguistic Feature Importance

We used the attribute of the extra trees algorithm to output the feature importance of the language features that were used for modeling. The importance of a feature indicates how important the feature was to the model building process. The sum of the values of the importance of the features used was 1. All 120 linguistic features were used to train the prediction model. As shown in Table 3, the sum of the importance of each lexicon was calculated. Since total feature importance increases with the number of features, we also calculated the average feature importance for each lexicon, which is the ratio of the sum of the importance of each lexicon compared with the number of features included in each lexicon (see Table 3).

The results of this study showed that the SC-LIWC made the greatest contribution to predicting PWB, reaching a total importance of more than 70%. However, with respect to average feature importance, the word-average importance of the SC-LIWC lexicon was the lowest. The linguistic features of CVD performed the best during the model building process, followed by CSD and MMD and then by Weibo-5BML and MFD. The levels of importance of the features of these 5 lexicons were higher than that of SC-LIWC.

**Table 3:** Feature importance of the different lexicons.

Lexicon	Total feature importance	Average feature importance
MFD^a^	0.095	0.00865
MMD^b^	0.018	0.00885
CVD^c^	0.019	0.00961
CSD^d^	0.116	0.00892
Weibo-5BML^e^	0.044	0.00872
SC-LIWC^f^	0.708	0.00814

^a^MFD: Moral Foundations Dictionary.

^b^MMD: Moral Motivation Dictionary.

^c^CVD: Culture Value Dictionary.

^d^CSD: Chinese Suicide Dictionary.

^e^Weibo-5BML: Weibo Basic Mood Lexicon.

^f^SC-LIWC: Simplified Chinese version of the Linguistic Inquiry and Word Count.

### Correlations of the Actual PWB Scores

The Pearson correlation coefficient *r* can describe the degree of linear correlation between 2 variables; *r*’s absolute value stands for the strength of the correlation. Table 4 shows that all the dimensions of the actual scale scores exhibited a significant positive correlation.

**Table 4:** Pearson correlations for each dimension (n=1427).

Dimensions				Prediction model													Scale by Ryff and Keyes [23]										
				EM_1_^a^		PR_1_^b^		AU_1_^c^		PG_1_^d^		SA_1_^e^		PL_1_^f^		EM_2_			PR_2_		AU_2_		PG_2_		SA_2_		PL_2_
**Prediction model**																											
	**EM_1_**																										
		*r*	1		0.51^g^		0.40^g^		0.14^g^		0.65^g^		0.38^g^		0.49^h^			0.30^i^		0.23^i^		0.10^i^		0.37^i^		0.22^i^	
		*P* value	—^j^		<.001		<.001		<.001		<.001		<.001		<.001			<.001		<.001		<.001		<.001		<.001	
	**PR_1_**																										
		*r*	0.51^g^		1		0.14^g^		0.22^g^		0.49^g^		0.28^g^		0.30^i^			0.49^h^		0.08^i^		0.12^i^		0.29^i^		0.16^i^	
		*P* value	<.001		—		<.001		<.001		<.001		<.001		<.001			<.001		.002		<.001		<.001		<.001	
	**AU_1_**																										
		*r*	0.40^g^		0.14^g^		1		0.19^g^		0.41^g^		0.36^g^		0.21^i^			0.07^i^		0.54^h^		0.12^i^		0.23^i^		0.20^i^	
		*P* value	<.001		<.001		—		<.001		<.001		<.001		<.001			.007		<.001		<.001		<.001		<.001	
	**PG_1_**																										
		*r*	0.14^g^		0.22^g^		0.19^g^		1		0.20^g^		0.51^g^		0.12^i^			0.12^i^		0.12^i^		0.49^h^		0.13^i^		0.30^i^	
		*P* value	<.001		<.001		<.001		—		<.001		<.001		<.001			<.001		<.001		<.001		<.001		<.001	
	**SA_1_**																										
		*r*	0.65^g^		0.49^g^		0.41^g^		0.20^g^		1		0.42^g^		0.35^i^			0.28^i^		0.23^i^		0.12^i^		0.50^h^		0.23^i^	
		*P* value	<.001		<.001		<.001		<.001		—		<.001		<.001			<.001		<.001		<.001		<.001		<.001	
	**PL_1_**																										
		*r*	0.38^g^		0.28^g^		0.36^g^		0.51^g^		0.42^g^		1		0.23^i^			0.16^i^		0.22^i^		0.28^i^		0.24^i^		0.49^h^	
		*P* value	<.001		<.001		<.001		<.001		<.001		—		<.001			<.001		<.001		<.001		<.001		<.001	
**Scale by Ryff and Keyes**																											
	**EM_2_**																										
		*r*	0.49^h^		0.30^i^		0.21^i^		0.12^i^		0.35^i^		0.23^i^		1			0.48^g^		0.35^g^		0.22^g^		0.61^g^		0.35^g^	
		*P* value	<.001		<.001		<.001		<.001		<.001		<.001		—			<.001		<.001		<.001		<.001		<.001	
	**PR_2_**																										
		*r*	0.30^i^		0.49^h^		0.07^i^		0.12^i^		0.28^i^		0.16^i^		0.48^g^			1		0.22^g^		0.27^g^		0.50^g^		0.34^g^	
		*P* value	<.001		<.001		.007		<.001		<.001		<.001		<.001			—		<.001		<.001		<.001		<.001	
	**AU_2_**																										
		*r*	0.23^i^		0.08^i^		0.54^h^		0.12^i^		0.23^i^		0.22^i^		0.35^g^			0.22^g^		1		0.22^g^		0.35^g^		0.29^g^	
		*P* value	<.001		.002		<.001		<.001		<.001		<.001		<.001			<.001		—		<.001		<.001		<.001	
	**PG_2_**																										
		*r*	0.10^i^		0.12^i^		0.12^i^		0.49^h^		0.12^i^		0.28^i^		0.22^g^			0.27^g^		0.22^g^		1		0.19^g^		0.47^g^	
		*P* value	<.001		<.001		<.001		<.001		<.001		<.001		<.001			<.001		<.001		—		<.001		<.001	
	**SA_2_**																										
		*r*	0.37^i^		0.29^i^		0.23^i^		0.13^i^		0.50^h^		0.24^i^		0.61^g^			0.50^g^		0.35^g^		0.19^g^		1		0.36^g^	
		*P* value	<.001		<.001		<.001		<.001		<.001		<.001		<.001			<.001		<.001		<.001		—		<.001	
	**PL_2_**																										
		*r*	0.22^i^		0.16^i^		0.20^i^		0.30^i^		0.23^i^		0.49^h^		0.35^g^			0.34^g^		0.29^g^		0.47^g^		0.36^g^		1	
		*P* value	<.001		<.001		<.001		<.001		<.001		<.001		<.001			<.001		<.001		<.001		<.001		—	

^a^EM: environmental mastery.

^b^PR: positive relations with others.

^c^AU: autonomy.

^d^PG: personal growth.

^e^SA: self-acceptance.

^f^PL: purpose in life.

^g^Correlation matrices that measure different traits using the same method.

^h^Correlation coefficients that use different methods to measure the same traits.

^i^Correlation matrices that use different methods to measure different traits.

^j^Not applicable.

### Structural Validity

Table 4 presents the matrix of the Pearson correlation coefficients between variables. The subdimensions of PWB were included in the multitrait-multimethod matrix, including environmental mastery, positive relations with others, autonomy, personal growth, self-acceptance, and purpose in life, and 2 methods were involved in this process, including the PWB subscales and the linguistic prediction model.

Structural validity includes 2 different aspects, specifically discriminant validity and convergent validity. In Table 4, the correlation coefficients using different methods to measure the same traits are all greater than the other numbers, which indicates that the convergent validity of our multiobjective prediction model is good. However, the discriminant validity of this model is fair. Except for the dimension of autonomy, not all the correlation coefficients using different methods to measure the same traits are greater than the corresponding coefficients using the same method to measure different traits.

### Criterion Validity

The Pearson correlation coefficients between the effective standard and the predicted scores of the “whole” data are shown as R_1_ in Table 5. When measuring a psychological variable using different assessment instruments or methods, the correlation coefficient between different instruments or methods is typically approximately 0.39 to 0.68 [51]. Our results are all significantly greater than or equal to 0.49, which means that the developed model exhibits good criterion validity.

**Table 5:** Criterion validity and split-half reliability of each dimension (n=1427).

Dimension	R_1_^a^	*P* value	R_2_^b^	*P* value
Environmental mastery	0.49	<.001	0.80	<.001
Positive relations with others	0.49	<.001	0.65	<.001
Autonomy	0.54	<.001	0.85	<.001
Personal growth	0.49	<.001	0.69	<.001
Self-acceptance	0.50	<.001	0.72	<.001
Purpose in life	0.49	<.001	0.80	<.001

^a^R_1_: Pearson correlation coefficients between the predicted scores of the “whole” data and the ground truth data of each dimension.

^b^R_2_: Pearson correlation coefficients between the predicted scores of odd “split-half” data and the predicted scores of even “split-half” data.

### Split-Half Reliability

The Pearson correlation coefficient of the predicted scores for the 2 sets of “split-half” data are displayed in Table 5 (see R_2_), with all dimensions reaching the level of significance, which suggests that the model has good split-half reliability.

## Discussion

### Principal Findings

This study tested the ability of the prediction model to predict well-being levels based on linguistic data drawn from social media. We collected PWB scale scores as the output, and based on 6 psychological lexicons, we extracted linguistic features of posts on Weibo as the input. We then developed a multiobjective prediction model featuring 6 dimensions. Subsequently, the importance of the 6 lexicons used for model building was analyzed. At last, we explored the linguistic prediction model's criterion validity, structural validity (both to verify the model’s validity), and split-half reliability (to verify the model’s reliability). The results suggest that the prediction model had good criterion validity and split-half reliability. However, when it comes to structural validity, the convergent validity of the model was really good, but the discriminant validity was not satisfactory.

In this study, we used domain knowledge to extract features, which increases the interpretability of this prediction model. The results of this study showed that the SC-LIWC had the highest contribution to predicting PWB, with a total importance of more than 70%. This finding is consistent with those in several previous studies that used LIWC as a closed-vocabulary method of extracting social media features [15,47,52] and provides evidence of the validity of SC-LIWC [31]. However, regarding the average importance of these 6 lexicons, the word-average importance of the SC-LIWC lexicon was the lowest. We speculate that this deficiency is due to the fact that SC-LIWC is a general psycholinguistic lexicon. In fact, we were able to use SC-LIWC to distinguish people’s emotional states, intentions, thinking styles, and individual differences, but SC-LIWC is not a targeted measurement of PWB nor other psychological characteristics associated with PWB. Therefore, like our hypothesis, the linguistic features extracted using the other 5 lexicons based on domain knowledge performed better [52,53]. We also found that the linguistic features of CVD performed the best. One possible explanation for this finding is that people cannot define mental health in isolation from culture [5]. PWB, as an important component of well-being, cannot be explained by theories that lack reference to culture [54]. Furthermore, all understanding of PWB is based on moral visions, which also explains the good performance of MMD and MFD [54]. In addition, our finding concerning the good performance of CSD is also in line with those of previous research that reported a significant negative correlation between people's PWB level and their suicidal attitudes [38]. As suggested by Gubler et al [55], this study investigated the correlation between emotion and PWB using Weibo-5BML to extract features and reported primary evidence.

Consistent with previous research pertaining to the prediction of well-being based on social media [15-17], the high criterion validity suggests that users’ PWB could also be identified solely based on social media posts. In addition, even if some state-of-the-art methods, such as deep learning [56] and open-vocabulary methods [19], were not used in this investigation, the prediction model could achieve better accuracy by relying solely on domain knowledge. Another finding was that all 6 dimensions of PWB had similar criterion validity. One possible explanation for this fact is that these 6 dimensions are all distinct components of positive psychological functioning and serve as a theoretical foundation for generating a multidimensional model of well-being [23,57]. Another such reason is the fact that we chose a multiobjective regression algorithm to preserve the correlations among PWB dimensions.

All dimensions also exhibited fairly high convergent validity. Specifically, the correlations between different approaches measuring the same trait were all stronger than the correlations between different approaches measuring different traits. As we would expect, this suggested that the 2 methods measured the same trait. Numerous earlier studies have confirmed that diverse types of mental health status are expressed in various ways in language and even the same type of mental health status also has different expressions in language at different levels [15,35,58]. Our finding indicates that different components of PWB may have specific expressions in language that are more detailed and refined. These unique expressions of each component should be explored in the future. Furthermore, given the strong correlations (0.24-0.85) among the scores of the subscales in the scale by Ryff and Keyes [23] and the strong correlations (0.19-0.61) between the predicted scores, it is reasonable the model failed to exhibit good discriminant validity.

Referring to the reliability, our results suggest a good level of split-half reliability across all dimensions (ranging from 0.65 to 0.85), which is similar to results (ranging from 0.66 to 0.88) reported in earlier studies that have examined the PWB scale by Ryff and Keyes [57,59,60]. The believable reliability result indicates that the linguistic features we extracted based on domain knowledge, rather than other random factors, can illustrate people’s psychological status to a certain extent. According to studies that have argued for the robustness of using social media to reflect people’s implicit traits [22,61], such variations may stem from other personal characteristics.

This study validated the predictive ability and reliability of machine learning models in the context of ground truth mental health data, which is an important issue that must be addressed. Another unexpected finding of this study is that our results confirmed that the application of specific domain knowledge could help improve the predictive performance of machine learning models and contribute to the interpretability of such models. Although closed vocabulary analysis was proved not as good as open vocabulary analysis in a previous study [19], our model still achieved relatively advanced prediction accuracy [62]. This also proves the importance of introducing domain knowledge training prediction models from the side. The prediction model trained in this study incorporated knowledge from experts in the form of psychological lexicons that depict the linguistic patterns associated with different levels of PWB, which improved the model performance. It should be noted that the current prediction model still cannot replace scales nor dictate the judgment of a counselor but instead provides alerts and decision-making support. However, our study still has positive implications for practical applications in nonprofessional settings. Specifically, because the method of measuring mental health using social media data has been endorsed by our findings, it could be used for self-examination, helping to gain a clearer picture of oneself. In addition, the method could also be used for large-scale user studies to quickly provide feedback on the psychological state of the public, which is of great help to social governance.

### Limitations

A limitation of this study is that most of our participants were young women. The participants in this study were randomly sampled from social media, which might have caused some degree of sampling bias. In addition, this study was only carried out in the Chinese Weibo online environment, so the findings cannot be generalized to other languages or online platforms. Last, the performance of the prediction model tested in a new data set is unknown.

### Future Work

In this study, we used the closed vocabulary method and a machine learning algorithm to develop our prediction model. Utilizing deep learning algorithms for natural language processing may bring some better outcomes, but this aspect was not the focus of this study. We could further combine domain knowledge and a deep learning method in the future. In addition, considering that the quality of the online questionnaire depends on the participants' conscious compliance with the rules for completing the questionnaire, we chose the 18-item version of the PWB scale by Ryff and Keyes to reduce the time required to complete the scale and ensure participants’ attention. Further studies could choose other scales to measure individuals’ well-being more precisely. For example, considering the discriminant validity was not satisfactory in this work, using scales (eg, Oxford Happiness Inventory) that combine different dimensions to a single target is also worth trying. In addition, future work could compare the prediction performance between a single additive target and multiple targets. Last but not the least, many studies have used prediction models to compare changes before and after in the same individuals [58,63]. These studies, including this study, all have a common assumption; that is, individuals before and after a certain time node are regarded as different individuals. This may not be sufficiently accurate to measure people’s longitudinal changes. Further work needs to be done to update the data set by merging longitudinal data into cross-sectional data; then, this more comprehensive data set could be used to build prediction models so as to improve the prediction accuracy within participants.

### Conclusions

This study proposed a linguistic prediction model based on social media. By confirming the validity and reliability of the model, this study verified the predictive power of social media corresponding to ground truth mental health data from the perspective of PWB. Our study has positive implications for the use of social media to predict mental health in nonprofessional settings such as self-monitoring or large-scale user studies. Future research can explore natural language processing methods combined with domain knowledge to enhance the interpretability of deep learning models.

## Abbreviations

CSD: Chinese Suicide Dictionary
CVD: Culture Value Dictionary
LIWC: Linguistic Inquiry and Word Count
MFD: Moral Foundations Dictionary
MMD: Moral Motivation Dictionary
MOET: multi-objective extra trees
PWB: psychological well-being
SC-LIWC: Simplified Chinese version of the Linguistic Inquiry and Word Count
Weibo-5BML: Weibo Basic Mood Lexicon

## Data Availability Statement

The data sets generated for this article are not readily available because the raw data cannot be made public; if necessary, feature data can be provided. Requests to access the data sets should be directed to the corresponding author.
